# Metastatic Crohn's Disease of the Ear

**DOI:** 10.1155/2009/871567

**Published:** 2009-03-16

**Authors:** J. H. Chuah, D. S. Kim, C. Allen, L. Hollis

**Affiliations:** ^1^Department of Otolaryngology and Head and Neck Surgery, Worcestershire Royal Hospital, Worcester WR5 1DD, UK; ^2^Department of Pathology, Worcestershire Royal Hospital, Worcester WR5 1DD, UK

## Abstract

*Objective*. We reported a very rare case of metastatic Crohn's disease involving the retro-auricular region. *Method*. A case report and a review of literature concerning metastatic Crohn's disease. *Results*. Metastatic Crohn's disease is an uncommon extraintestinal cutaneous manifestation of Crohn's disease and a very rare case involving the retro-auricular region is reported here. Given the limited existing literature little is known about this condition. The skin lesions appear to have a predilection for the lower trunk and genitalia regions. There is no clear association with the severity of Crohn's disease and in some cases, the cutaneous lesions predate the onset of gastrointestinal Crohn's disease. Treatment with immune-modulating medications together with the antitumour necrosis factor monoclonal antibody therapy appears to offer the best chance of remission. *Conclusion*. By reporting this interesting and rare condition we also hope to highlight the importance of considering underlying chronic systemic disorders, such as Crohn's disease, when presented with skin lesions resistant to simple local treatments.

## 1. Introduction

Metastatic cutaneous Crohn's disease is a rare manifestation of Crohn's disease. Its hallmark features include the presence of cutaneous granulomatous lesions noncontiguous with the gastrointestinal tract or fistulae [1]. The clinical appearance can vary and biopsy is required to confirm the diagnosis.

We report a very rare case of metastatic Crohn's disease affecting the ear in a man with longstanding Crohn's disease. We aim to highlight the need for clinical suspicion of systemic diseases that may present as a localised ear lesion, in particular, the rare entity of metastatic Crohn's disease.

## 2. Case Report

A 43-year-old man with an 18 years history of Crohn's disease developed a severe erythematous and exudative skin lesion in the right postauricular cleft region. Prominent purulent discharge and microabscesses suggested an infective lesion. The patient had recently completed a course of the therapeutic drug infliximab, a monoclonal antibody for tumour necrosis factor (TNF), which is a key inflammatory agent in systemic inflammatory conditions. Gastroenterologists were concerned that the skin lesion was an infective complication of the new therapeutic regimen due to a possible immunosuppressive action. Several antibacterial agents were used with no clinical benefit, and the patient was then referred to the ENT department for a second opinion. Concurrently, the patient had a recurrence of severe perineal Crohn's disease and warranted further course of infliximab. However, given the issue of possible association with the skin lesion, the treatment decision was deferred pending resolution of the skin condition.

Initial ENT examination excluded an otitis externa causing local inflammation from overspill onto the adjacent skin area. Appearance of the skin lesion was in keeping with either an infection or severe eczematous inflammation. A skin swab for culture was taken and triadcortyl ointment applied. During the initial consultation, the patient complained of a similar lesion starting in the umbilicus region. The swab result reported heavy growths of beta-hemolytic streptococcus and *Staphylococcus aureus* with moderate anaerobes. Triple therapy with flucloxacillin, erythromycin, and metronidazole proved to be ineffective. During this period, there was gradual deterioration of the lesion, which prompted a biopsy of the periauricular skin lesion. The pathologist reported the biopsy to represent a chronic noncaseating granulomatous lesion consistent with a diagnosis ofmetastatic Crohn's disease (please refer pathology report).

Subsequently, with dermatology advice, treatment with high-dose betnovate cream, oral steroids, and infliximab resulted in rapid remission of the skin condition (both the postaural and umbilical areas) and the perineal Crohn's disease.

## 3. Pathology Report

**Figure 1:**
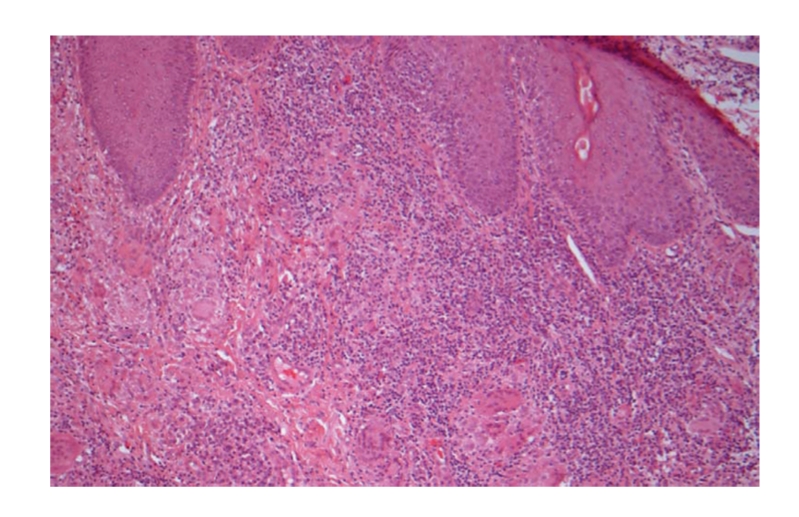
Skin biopsy (x10 objective, haematoxylin, and eosin stain) showing a dermal chronic inflammatory cell infiltrate with noncaseating epithelioid and giant cell granulomas. Although there are no pathognomonic features, this is consistent with metastatic Crohn's disease.

Histological haematoxylin and eosin sections (Figure 1) demonstrated chronic inflammation of the dermis with admixed epithelioid and giant cell granulomas. Schaumann's bodies were seen within the giant cells. There was no caseation, and elastic fibres were absent from the granulomas. Furthermore, there was an absence of fungi, staining for acid and alcohol fast bacilli were negative, and birefringent foreign bodies were not seen. These appearances were reported as consistent with cutaneous Crohn's disease.

## 4. Discussion

Metastatic cutaneous Crohn's disease refers to the presence of a skin lesion containing epithelioid and giant cell granulomas as seen in the affected bowel segments of patients with Crohn's disease, but is physically separated from the gastrointestinal tract [2]. This condition is distinct from other more well-characterized cutaneous manifestations which include pyoderma gangrenosum, erythema nodosum, polyarteritis nodosa, and epidermolysis acquisita. Although the estimated incidence of cutaneous manifestation of Crohn's disease varies widely between 2% and 44% [3], true metastatic cutaneous Crohn's disease is exceedingly rare.

Metastatic Crohn's disease does not appear to have any distinct relationship with the severity of the Crohn's bowel disease [3], and indeed may even precede gastrointestinal involvement [3]. However, there is a suggestion in the limited literature available that there may be an association with perianal Crohn's disease [3]. Of the reported cases of metastatic Crohn's disease involvement primarily of the lower limbs and trunk regions with a predilection for skin fold areas and genitalia has been described [4]. There has been only one reported case of facial involvement documented more than 30 years ago.

To date, given the rarity of the condition, there is no gold-standard therapy for metastatic Crohn's disease [2]. The use of systemic and topical steroids, antibiotics, and immunosuppressive agents such as azathioprine, sulfasalazine, and methotrexate has been described with variable success [4]. The use of Infliximab, an antitumour necrosis factor monoclonal antibody, as one of the latest treatment options has been described in a few case reports to be more effective in maintaining disease remission, especially with repetitive administration and when used concomitantly with other more mainstream therapies [5–7]. Further evidence is needed to substantiate this. However, given their recent introduction to clinical use, in addition to a better evidence for clinical efficacy in various disorders, new side effects may also become apparent with time and should be carefully evaluated.

In the case being presented, the possibility of an unusual diagnosis for the skin lesion was raised by several features: the sudden onset of postauricular rash in a gentleman with no previous ear or skin condition; the aggressive nature of the purulent rash; the observed resistance to several common treatments used including multiple antimicrobial agents and topical steroid cream. The diagnosis of the rare cutaneous Crohn's disease was first suggested by the biopsy demonstrating chronic granulomatous inflammation in a patient known to be suffering from active Crohn's disease. Biopsies demonstrating chronic granulomatous inflammation of the skin require the pathologists to consider other and more common disorders including sarcoidosis, tuberculosis and fungal or parasitic infections, foreign body granulomas, and annular elastocystic granuloma. Histological analysis failed to demonstrate any of the pathognomonic features of the other granulomatous conditions. The diagnosis of metastatic Crohn's was further supported by the rapid remission both of the skin rash and perineal Crohn's disease in response to treatment with high-dose local steroid cream, oral steroids, and concomitant infliximab infusion.

Despite its rarity, a meticulous evaluation including a thorough history and examination, and biopsy in cases unresponsive to simple measures, together with a high index of clinical suspicion in those with an established diagnosis or presenting with concomitant clinical features of Crohn's disease, may provide early diagnosis of this skin condition which can be disfiguring and emotionally distressing to the patient. The diagnosis of metastatic Crohn's disease may be relatively easier in patients with established Crohn's disease compared to those cases where the skin lesions precede bowel involvement. In the latter cases, diagnosis by biopsy should then lead to appropriate counseling about the diagnosis of Crohn's disease and the future onset of the more significant bowel disease.

We hope by reporting this rare and interesting case that we may highlight the need to consider systemic diseases, in particular, chronic inflammatory conditions such as Crohn's disease, as causes of skin lesions of the ear and face that are unresponsive to simple treatments.

## 5. Summary

- Metastatic Crohn's disease is an uncommon cutaneous manifestation of Crohn's disease.
- Metastatic Crohn's lesions have a predilection for the skin fold, limbs, and trunk.
- Metastatic Crohn's involving ear is extremely rare.
- To date, there is no gold standard treatment for metastatic Crohn's disease.
- Treatment with repetitive administration of Infliximab together with other mainstream therapies is promising in maintaining disease remission.
